# City Dwellings for the Working Classes

**Published:** 1857-10

**Authors:** 


### CITY DWELLINGS FOR THE WORKING CLASSES.

Mr. Ross, in the Court of Common Council of the City of London, has been conducting an able argument on the best mode of providing dwellings for the working classes.

Mr. Ross shows with great force and exactness that the poor population of London is increasing, and that the idea of securing suburban villages for these poor classes is hopeless. He proposes, therefore, the erection of suitable buildings in the City itself. Calculating on the scheme of the metropolitan association, he finds that 20,000 persons could be accommodated on eight acres of ground. According to his plan, on proportionately less space, or about six and a half acres, and allowing as much space for yards as would be occupied with buildings, thirteen acres would suffice to house this large number of persons. The City contains 640 acres. Surely 13 of these could be, and ought to be, spared to provide houses for its working people.

Was ever argument more impressive or simple? Yet imagine that in the assembly where it was spoken the speaker stood almost alone in the cause, the one representative there of scientific philanthropy. We shall return to this subject. Meantime we need not urge Mr. Ross to push on in his labours; he grasps the work, understands the delay, and sees the reward.

